# 0083. Hepatoprotective effects of hydrogen sulphide against acute liver failure

**DOI:** 10.1186/2197-425X-2-S1-P3

**Published:** 2014-09-26

**Authors:** K Tokuda, F Ichinose

**Affiliations:** Kyushu University Hospital, Intensive Care Unit, Fukuoka, Japan; Massachusetts General Hospital, Department of Anesthesia, Critical Care and Pain Medicine, Charlestown, MA USA

## Introduction

Acute liver failure is a fatal syndrome attributed to massive hepatocyte apoptosis that is resistant to conventional medical therapies. Consequently, liver transplantation is required in many cases. An experimental liver failure model induced by galactosamine (Gal) and lipopolysaccharide (LPS) mimics clinical acute liver failure. In this model, LPS stimulates macrophages to release TNFα, which induces apoptosis in Gal-sensitized hepatocytes, causing acute liver failure. Hydrogen sulphide (H_2_S), which is an endogenously produced gaseous signaling molecule, has anti-apoptotic as well as anti-inflammatory properties. Previously, we reported that H_2_S attenuates liver dysfunction arising from LPS-induced systemic inflammation [1]. It has also been reported that H_2_S reduces hepatic ischemia/reperfusion injury by inhibition of apoptosis in the liver [2]. However, it is still unknown whether H_2_S exerts hepatoprotective effects against acute liver failure, in which both inflammatory responses and apoptosis have critical roles. Here, we examined the impact of H_2_S on acute liver failure in mice induced by Gal and LPS.

## Methods

Mice were challenged with saline or combination of Gal and LPS intraperitoneally, then divided into two groups: one group breathed air alone, and another breathed H_2_S (80 ppm) for 6h followed by breathing air.

## Results

Mice that breathed air after Gal/LPS challenge showed poor survival rate (13%) and marked increase of alanine aminotransferase (ALT) and aspartate aminotransferase (AST) in plasma. On the other hand, H_2_S inhalation for 6h after challenge markedly improved survival (60%, *p* < 0.05) and suppressed Gal/LPS-induced elevation of ALT and AST levels in plasma. Inhaled H_2_S suppressed TNFα in plasma at 1h after Gal/LPS challenge. Mice that breathed air after Gal/LPS challenge exhibited activation of caspase 3, 8, and 9 in the liver, whereas H_2_S breathing inhibited activation of caspase 3, 8, and 9, suggesting inhaled H_2_S after Gal/LPS challenge suppressed both extrinsic and intrinsic pathways of caspase-dependent apoptosis in the liver. Gal/LPS challenge increased phosphorylated STAT3 transcription factor. H_2_S inhalation after Gal/LPS challenge further augmented phosphorylation of STAT3 compared to air alone. The protective effects of H_2_S inhalation after Gal/LPS challenge were associated with upregulation of gene expression of anti-inflammatory IL-10, which stimulates STAT3 phosphorylation, in the liver. These results suggest that inhaled H_2_S contributes to survival of mice in acute liver failure at least in part through activation of IL-10/STAT3 pathway.

## Conclusions

These results suggest that H_2_S shows hepatoprotective effects against acute liver failure at least in part by inhibition of caspase activation and by augmentation of IL-10/STAT3 signaling pathway in the liver.
